# Genetic diversity and phylogenetic analysis of native mountain ponies of Britain and Ireland reveals a novel rare population

**DOI:** 10.1002/ece3.507

**Published:** 2013-03-05

**Authors:** Clare L Winton, Matthew J Hegarty, Robert McMahon, Gancho T Slavov, Neil R McEwan, Mina CG Davies-Morel, Charly M Morgan, Wayne Powell, Deborah M Nash

**Affiliations:** IBERS, Aberystwyth UniversityAberystwyth, Ceredigion, SY23 3DA, UK

**Keywords:** Conservation, microsatellite, mtDNA, phylogenetics, SNP, Welsh pony

## Abstract

The conservation of unique populations of animals is critical in order to preserve valuable genetic diversity and, where populations are free-living, maintain their irreplaceable influence upon habitat ecology. An accurate assessment of genetic diversity and structure within and between populations is crucial in order to design and implement conservation strategies in natural and domesticated species. Moreover, where it is possible to identify relic populations that are related to a structured breed an ideal opportunity presents itself to model processes that reveal historical factors that have shaped genetic diversity. The origins of native UK mountain and moorland ponies are uncertain, but they may have directly descended from prehistoric populations and potentially harbour specific adaptations to the uplands of Britain and Ireland. To date, there have been no studies of population structure and genetic diversity present within a free-living group of ponies in the Carneddau mountain range of North Wales. Herein, we describe the use of microsatellites and SNPs together with analysis of the mitochondrial control region to quantify the extent and magnitude of genetic diversity present in the feral Carneddau pony and relate this to several recognised British and Irish pony breeds. Our results establish that the feral Carneddau ponies represent a unique and distinctive population that merits recognition as a defined population and conservation priority. We discuss the implications for conservation of this population as a unique pool of genetic diversity adapted to the British uplands and potentially of particular value in maintaining the biodiversity of these habitats.

## Introduction

An understanding of the genetic characteristics of a population is paramount in order to inform and implement conservation strategies that preserve and maintain genetic diversity (Hall and Bradley 1995). This principle applies to both domestic livestock breeds and wild species in situ. Bruford et al. (2003) highlighted the complexity of domestication and a need to better understand the population dynamics involved to guide livestock conservation and maintain biodiversity resources for the future. Domestication of livestock has resulted in the selection of certain animals according to their suitability for specific tasks, leading to post-domestic polychotomy in phenotypic appearance within species. Breed development occurs by the mixing of different populations, or by selecting from a sub-population within an existing breed, to create a group of animals that contain a defined set of desired traits (Goodwin et al. 2008).

Several definitions of what constitutes a breed exist, and the term is therefore flexible and potentially confusing dependent upon the context in which it is used. Clutton-Brock (1987) defines a breed as “a group of animals that has been selected by humans to possess uniform appearance that is inheritable and distinguishes it from other groups of animals within the same species.” Lush and Crow (1994) argues that the term “breed” was created by breeders of livestock prior to the recognition of genetic selection and is commonly used in lay-situations according to popular consensus. In practice, certain breed societies will not register an individual within a breed stud book without a pedigree demonstrating several generations of closed matings (Welsh Pony and Cob Society). Other societies register animals according to both pedigree and morphological assessment of phenotype based upon agreed guidelines (Connemara Society). Nonetheless, the issue of whether or not a group of individuals can be designated as a certain breed should not detract from the recognition that a particular population, with a distinct genetic profile following substantial isolation, may be worthy of conservation.

A better understanding of population origin and history of development gained through molecular genetic analysis may provide information on relic populations. Putative genetic links may then be made to ancestral groups from which modern breeds have developed following periods of population management (Hall and Bradley 1995). Molecular approaches have begun to be applied to the genetic examination of feral populations of horses around the world in comparison to managed registered breeds that show phenotypic and/or geographical similarities (Vega-Pla et al. 2006; Plante et al. 2007; Rendo et al. 2012). The primary aims of these studies were to identify conservation priorities and guide conservation management schemes. In Britain, there are only a limited number of free-living populations of domesticated livestock species (Hall and Moore 1986; Goulding 2001; Wilson 2003; Burthe et al. 2011), and to-date no molecular genetic analysis has been conducted upon semi-feral British native pony breeds with a view to better understand the structure of genetic diversity.

**Figure 1:**
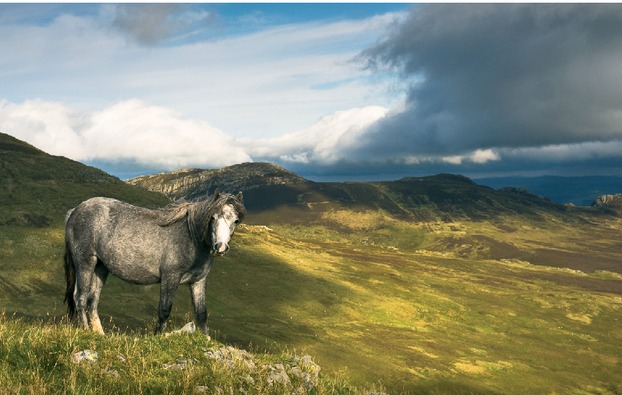
The free-living Carneddau pony in its natural environment in north Wales. Photo courtesy: ©2012 Osian Rees.

A free-living equine population known as Carneddau ponies exist in Snowdonia National Park (Wales) that have been suggested to be direct descendants of the ancestral Welsh Mountain Pony (Fig. 1). The Carneddau ponies are anecdotally thought to have existed during the Roman era and have been implicated as representative of the historical foundation of a modern, registered breed, the Welsh Section A pony. Carneddau ponies are one of the last remaining collections of free-living ponies in Britain. Compared to other equine populations maintained on hills or moors, Carneddau ponies are less intensively managed in terms of reproduction, with intervention being limited to late autumn removal of a proportion of offspring for sale. Access to grazing, water and shelter, social interactions and general maintenance of these mares, stallions and residual young stock are unhindered by human intervention. The geographically nearest group of registered, traditionally managed animals to the Carneddau population is the Welsh Pony and Cob, a breed indigenous to Wales. The Welsh Pony and Cob is sub-divided into four distinct sections whose defining criteria are the animal's adult height and conformation characteristics: Section A is a Welsh Mountain Pony, <121.9 cm; Section B is a Welsh Riding Pony, of fine-built morphology, <137.2 cm; Section C is a Welsh Pony of Cob Type, of stocky morphology, <137.2 cm and Section D, a Welsh Cob and >137.2 cm (Davies 1993). The term “Cob” refers to a generically bred animal of stocky morphology, although in the case of the “Welsh Cob” it refers to registered animals of a defined phenotype (according to Welsh Pony and Cob Society guidelines). All four sections show phenotypic similarities and are assumed to have arisen from a common ancestral stock similar to the modern Section A. The Welsh Pony and Cob Society (WPCS) administer a stud book for pedigree documentation of all four sections, which has been closed since 1950, meaning that offspring resulting from breeding with unregistered animals thereafter may not themselves be registered (Davies 2001).

No previous studies have examined Carneddau ponies in terms of effective population size, degree of inbreeding or their molecular relationship to Welsh and other UK and Ireland native pony breeds. The topography of Wales creates natural environmental barriers to gene flow and population migration in the form of mountain ranges and expanses of water. More specifically, the Carneddau ponies are limited to the Carneddau mountain range and are believed to have maintained a relatively stable population of around 300 breeding animals. Thus, the Carneddau may have evolved or maintained adaptations specific to their upland habitat that have been lost from the related ponies during breed formation. We present a comprehensive analysis of the genetic diversity within the Carneddau ponies relative to other UK native pony breeds from Wales, Ireland and Scotland. The aim is to establish to what extent they represent a genetically unique subpopulation formed as a result of long-term geographical isolation within Wales or whether this population is a more recent separation from the existing Section A pony breed.

## Materials and Methods

### Sample collection and extraction of genomic DNA

Studies were conducted using animals representative of different bloodlines for each of five British-Irish native pony populations. Samples were collected from Welsh Section A and Section D animals, Connemara ponies (native to Ireland) and Highland ponies (native to Scotland) from individuals registered with their respective breed society stud book (see Table 1 for sample numbers and population identifier abbreviations). In each case, collection from siblings was avoided. Samples were also collected from feral Carneddau ponies during a single annual herd round-up in 2009, although the relationship between these source animals was not known. All samples were collected as tail hairs or buccal swabs. Total DNA extraction from hair was performed using the Qiagen DNeasy® Blood and Tissue extraction kit (Qiagen, West Sussex, UK) according to manufacturer's instructions. Total DNA from buccal samples were extracted using the BuccalAmp™ DNA Extraction Kit (EPICENTRE® Biotechnologies, Cambio Ltd., UK).

**Table 1:** Summary statistics for MTCR, SSR and SNP genotyping for each of the five sample groups

Population (Identifier)	*N* (MTCR/SSR/SNP)	*H*	*h* ± SD	*π* ± SD	MNA (SSR/SNP)	*H*o (SSR/SNP)	*H*e (SSR/SNP)	*F*_IS_ (SSR/SNP)	*No. Markers* (SSR/SNP*)*
Section A (A)	47/48/12	17	0.932 ± 0.017	0.0179 ± 0.0093	7.47/1.86	0.677/0.276	0.726/0.275	0.0536/−0.001	17/48,703
Section D (D)	46/46/12	26	0.950 ± 0.025	0.0190 ± 0.0077	6.65/1.82	0.637/0.266	0.702/0.266	0.1015**/−0.003	17/48,715
Carneddau (S)	46/46/12	8	0.788 ± 0.0361	0.0146 ± 0.0077	6.94/1.84	0.659/0.282	0.733/0.272	0.0868**/−0.037	17/48,715
Connemara (N)	46/46/12	19	0.878 ± 0.032	0.0137 ± 0.0072	6.65/1.91	0.672/0.303	0.748/0.295	0.0922**/−0.029	17/48,716
Highland (H)	45/39/12	14	0.916 ± 0.019	0.0142 ± 0.0075	6.41/1.80	0.650/0.263	0.723/0.262	0.0320/−0.004	17/48,710
Total	225/233/60	53	0.932 ± 0.0171	0.018 ± 0.0090	9.53/1.84	0.659/0.278	0.778/0.304	0.0749**/0.086	17/48,327

*N =* sample number, MTCR = mitochondrial control region, SSR = simple sequence repeat, SNP = single nucleotide polymorphism, *H* = number of MTCR haplotypes, *h* = MTCR haplotype diversity, *π* = MTCR nucleotide diversity, MNA =mean number of alleles, *H*_O_ = observed heterozygosity, *H*_E_ = expected heterozygosity, *F*_IS_ = inbreeding coefficient, SD = standard deviation, *No. Markers* = number of (SSR/SNP) markers used in *H*_O_, *H*_E_ and *F*_IS_ calculations for each population.

*Significant deviation from HWE at *P <* 0.05.

**Significant deviation from HWE at *P* < 0.01.

### SSR genotyping and data analysis

Microsatellite (Simple Sequence Repeat; SSR) amplification by PCR was performed using the StockMarks® equine genotyping kit (Applied Biosystems, Warrington, UK) comprising 17 multiplexed fluorescent dye-labelled primers for the microsatellite markers, recommended by the International Society of Animal Genetics. PCR amplification used 10 ng of template genomic DNA with reagents at concentrations as provided; 2.5 μL Stockmarks PCR Buffer, 4 μL dNTP mix, 0.5 μL AmpliTaq Gold® DNA Polymerase, 4 μL multiplexed primer mix and 3 μL water. DNA was amplified in a G-Storm GS-1 thermal cycler (Gene Technologies Ltd., Essex, UK) and reactions incubated at 95°C for 10 min followed by 40 cycles of 95°C for 30 sec, 60°C for 30 sec and 72°C for 60 sec, followed by final extension at 72°C for 60 min. PCR products were diluted 1:20 with molecular grade water and subjected to capillary electrophoresis using an ABI 3730 DNA Analyzer (Applied Biosystems). Alleles were sized using the internal size standard GeneScan™ -500LIZ (Applied Biosystems). Size analyses of separated DNA fragments were performed with the GeneMapper software version 3.7 (Applied Biosystems). Alleles were scored based on PCR product size and recorded manually. Allele frequencies were examined for significant deviation from Hardy-Weinberg expectations using the exact test in Arlequin 3.5 (Excoffier et al. 2005; Excoffier and Lischer 2010), with Markov chain length set to 100,000 following 10,000 dememorization steps. Markers that demonstrated highly significant deviations in more than one population and/or contained large amounts of missing data values were removed to limit the influence of allele drop out. Loci with missing data unevenly distributed across the populations (>5% per breed) were also excluded to limit ascertainment bias. According to these criteria, five loci were removed from the analysis, including two that resulted in null alleles in other pony breeds (Rendo et al. 2012). Individual samples with poor quality DNA with calls missing from >3 loci were also excluded. To elucidate the relationships between the five populations and provide fine quantification of the different ancestral contributions, an unbiased Bayesian approach using Markov chain Monte Carlo (MCMC) clustering of samples was conducted via the STRUCTURE v2.2.3 software (Pritchard et al. 2000) using both SSR and SNP loci. Allele calls were loaded into STRUCTURE as diploid data for each individual and assessed for prior values of *K* ranging from 2 to 9. Burn-in and MCMC iteration settings were 50,000 and 10,000, respectively. Allele frequencies were treated as correlated. For each value of *K,* three replicate simulations were conducted. The Δ*K* statistic (the second order rate of change in log probability between successive values of *K*) was calculated using STRUCTURE Harvester v0.6.7 (http://taylor0.biology.ucla.edu/struct_harvest/) as per Evanno et al. (2005). Results from replicate runs at the optimal *K* were combined in CLUMPP (Jakobsson and Rosenberg 2007) and the average Q-table exported to DISTRUCT (Rosenberg 2004) for graphical presentation. Population summary statistics and analysis of molecular variance (AMOVA) were calculated using Arlequin 3.5 (Excoffier and Lischer 2010).

### Single Nucleotide Polymorphism (SNP) genotyping and data analysis

A subset of 12 individuals from each group was selected and 200 ng of genomic DNA was supplied to Central Biotechnology Services (Cardiff University) for genotyping using the Illumina Equine SNP50 assay. Data were returned as binary allele calls and reformatted for STRUCTURE. Data were filtered for quality control purposes by removing any loci with >10% missing data and/or loci with extreme deviation from HW expectations (*P* < 0.001) (determined using PLINK http://pngu.mgh.harvard.edu/purcell/plink/). After filtering observed heterozygosity (*H*_o_), expected heterozygosity (*H*_e_) and *F*_IS_ values were calculated using PLINK, using between 48,703 and 48,710 SNPs per population (see Table 1). The proportion of shared alleles identical by state (IBS) between individuals was calculated in PLINK and the resultant similarity matrix used to establish the relationships between the horses using UPGMA clustering in MEGA4 and by multidimensional scaling within PLINK. To further investigate any higher order patterns within the SNP data, STRUCTURE analysis was performed on three random subsamples of 5000 markers each analysed for values of *K* ranging from 2 to 10 (five iterations per *K*). As Alpha values stabilised rapidly, burnin and MCMC values were set at 25,000 each to improve computational speed. Data from each subsample analysis were combined and Δ*K* statistics calculated using STRUCTURE Harvester as for the SSR analysis. Results from replicate runs at the optimal *K* were combined in CLUMPP (Jakobsson and Rosenberg 2007) and the average Q-table exported to DISTRUCT (Rosenberg 2004) for graphical presentation.

### Mitochondrial DNA sequencing and analysis

Using a Welsh Cob sequence (GenBank AF072983), the primer pair (Forward - 5′-ATT TCT TCC CCT AAA CGA CAA C-3′) and (Reverse - 5′-CGT TCA ATT TAA GTC CAG CTT C-3′) was designed to a 606 bp amplicon of the mitochondrial control region (MTCR). PCR amplifications consisted of 10 ng template DNA, 7.5 μL of ImmoMix (Bioline, London, UK), 1 μmol/L forward and reverse primer and water to a final volume of 15 μL. Amplifications were performed in a G-Storm GS-1 thermal cycler at 95°C for 10 min, followed by 35 cycles of 95°C for 30 sec, 55°C for 30 sec and 72°C for 60 sec, with final extension at 72°C for 10 min. Reaction products were purified using a Multiscreen® 96-well filter plate (Millipore, Durham, UK) to remove primers and dNTPs then diluted 1:20 with molecular grade water in preparation for sequencing using an ABI 3730 DNA Analyzer (Applied Biosystems) and 1 μmol/L of the forward primer described above. To place the current data into a wider context against the major worldwide horse mtDNA haplogroups, 83 reference horse and pony samples were downloaded from GenBank based upon a recent whole mitochondrial genome study (Achilli et al. 2012) and included within the data file. Sequence data were trimmed to a common 519 bp sequence and aligned using BioEdit Sequence Alignment Editor v7.0.9.0 (Hall 1999) before being exported in phylip (phy.) format. The package NETWORK (http://www.fluxus-engineering.com) was employed to produce a median-joining network based on maximum parsimony to reconstruct the phylogenetic relationships among haplotypes. Individuals showing unusually long branch lengths as part of the network were examined more closely for poor sequence quality. Sequence traces were checked for nucleotide call errors and either manually rectified or removed in the case of overall low-quality sequence. Population allelic and molecular diversities were compared using Arlequin 3.5 (Excoffier and Lischer 2010).

## Results

### Population genetic diversity parameters

Summary statistics for MTCR, SSR and SNP data are presented in Table 1. Analysis of MTCR sequence from 230 individuals (*n* = 47 for Section A, *n* = 46 for Section D, *n* = 46 for Connemara, *n* = 45 for Highland and *n* = 46 for Carneddau) (Table 1) identified 54 haplotypes based on 60 variable nucleotide sites. Haplotype diversity was high (0.932 ± 0.0171) with relatively low nucleotide diversity (0.018 ± 0.0091). The Carneddau possessed the lowest haplotype diversity of the five groups (but not the lowest nucleotide diversity), and also the lowest number of haplotypes (approximately half that observed in the other groups). For SSRs, 225 individuals were successfully genotyped using the StockMarks kit (*n* = 48 for Section A, *n* = 46 for Section D, *n* = 46 for Connemara, *n* = 39 for Highland and *n* = 46 for Carneddau) (Table 1). The mean number of alleles per locus (MNA) ranged from 6.4 to 7.5 for the 17 SSRs, with average expected (*H*_E_) and observed (*H*_O_) heterozygosity values of 0.778 and 0.659 respectively. In contrast to the MTCR results, the Carneddau showed higher expected and observed heterozygosity than both the Highland and the Section D. The Section D samples possessed the lowest number of alleles, the lowest *H*_E_ and *H*_O_ and also the highest inbreeding coefficient (*F*_IS_), demonstrating a clear reduction in heterozygosity probably due to non-random mating. Section A showed the highest MNA although Connemara had equal *H*_O_ and displayed higher *H*_E_. SNP results, based on 12 individuals per population, show roughly similar patterns of diversity with no significant differences in heterozygosity between the 5 groups, but with the Connemara ponies again showing the highest expected heterozygosity (*H*_e_ = 0.342) and the Carneddau the highest observed (*H*_o_ = 0.338). Global exact tests of HWE performed across all loci revealed all five groups deviated significantly (*P* < 0.01) from HWE indicating a significant level of structure between the groups. Molecular analysis of variation demonstrated highly significant levels of between group variation relative to within group for both SSR and MTCR results, with 9% and 85% of total variation between groups respectively. Individual HW tests indicate that consistently significant deviations were found across several of the populations for a few of the SSR markers. These loci were removed from the data set for later STRUCTURE analysis below, to exclude the possibility of allele drop out. Similar results were obtained with SNP data again indicating that the Carneddau had relatively high heterozygosity levels and no evidence of significant inbreeding.

### Population structure and gene flow

**Figure 2:**
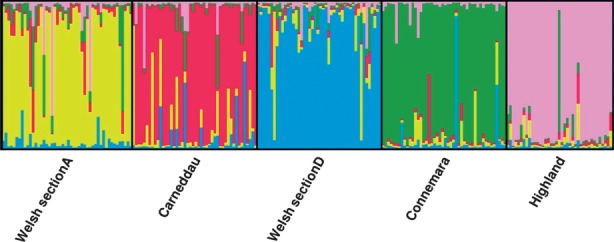
The results of STRUCTURE analysis for SSR loci of each population for *K* = 5. STRUCTURE Harvester analysis as per Evanno et al. (2005) suggests true value of *K* is 5. There is no real further subdivision beyond 5 clusters at *K* > 5.

**Figure 3:**
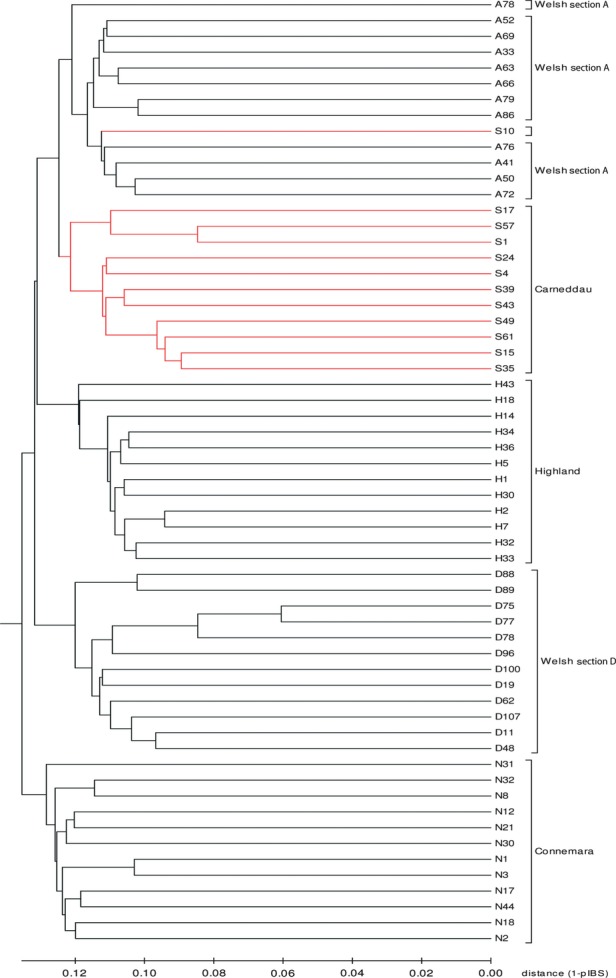
Clustering of genetic distance between individuals based on 1-proportion of alleles identical by state. The phylogeny was inferred using the UPGMA method (Sneath and Sokal 1973). The optimal tree with the sum of branch length = 6.66,442,597 is shown. The tree is drawn to scale, with branch lengths in the same units as those of the distances used to infer the phylogenetic tree. Phylogenetic analyses were conducted in MEGA4 (Tamura et al. 2007). The identity of individual horses and the groups they belong to are shown on the right.

**Figure 4:**
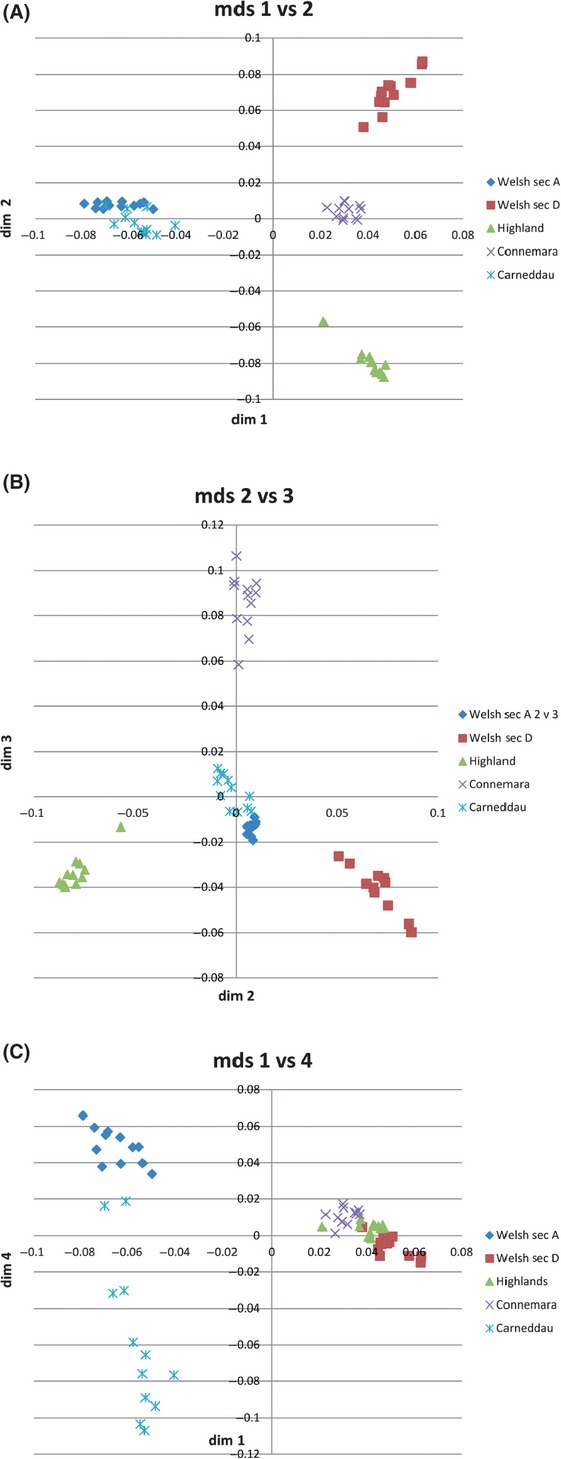
A–C. Identity by State similarity scores were calculated in PLINK from 48,721 diallelic markers. The resultant similarity between individuals was subjected to classical metric multidimensional scaling and the first four most significant dimensions extracted and used to plot the three graphs shown above. Dimension 1 splits Section A/Carneddau from the rest, Dimension 2 seperates Section D and Highlands from the rest, Dimension 3 seperates Connemaras from the rest and Dimension 4 seperates Carneddau and to a lesser extent Section A from the rest.

**Figure 5:**
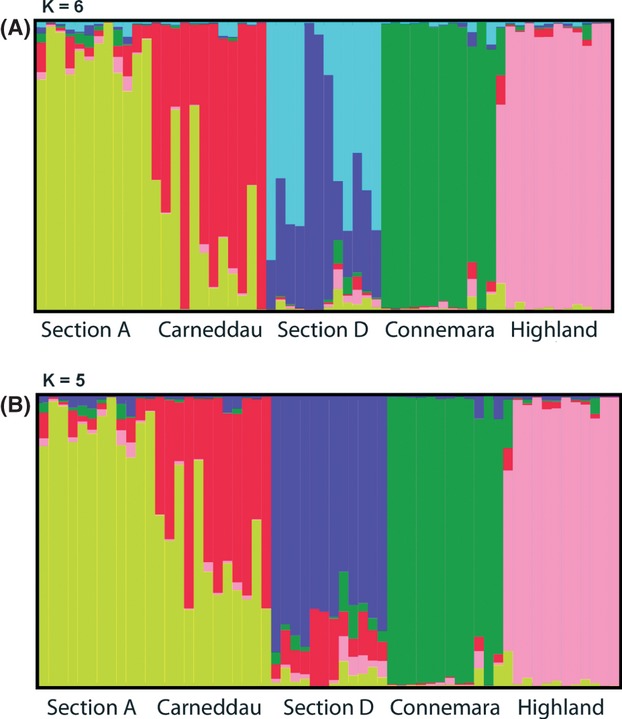
The results of STRUCTURE analysis for values of *K* = 6 (Fig. 5A) and *K =* 5 (Fig. 5B), averaged over three independent runs of 5000 SNPs and 12 ponies per group. STRUCTURE Harvester analysis suggests optimal cluster value of *K* is 6.

To elucidate the relationships between the five populations and provide finer quantification of the different ancestral contributions, STRUCTURE analysis was conducted using both SSR and SNP loci. Initial analysis included a small number of two other Welsh pony groups, Section B and Section C. These two categories are known to be intermediate between Section A and D ponies in terms of height and conformation and in fact the direct offspring of a Section A crossed with a Section D may be registered as a Section C. This is clearly demonstrated within the STRUCTURE plots obtained (Fig. A1). The partial assignment of most Section B/C animals to both Section A and Section D clusters confirmed the package was effectively detecting cases of known admixture. The Section B and Section C animals were excluded from the main analysis as the sample size available were much fewer than in the samples from the 5 populations whose results are presented here. STRUCTURE analysis of the log likelihood [Ln Pr(*X/K*)] of the posterior probability of the SSR data for a given *K* suggested the optimal value of *K* was 5. The five clusters that were assigned match closely to the populations from which individuals had been sampled, though all populations contained individual animals that could not be unambiguously assigned to a single cluster (Fig. 2). Cluster analysis based on SNP data (Figs. 3, 4) also clearly demonstrates the separation of individuals into groups corresponding to their “breed”. The Section A and the Carneddau clusters are generally closest in multidimensional space and in the hierarchical cluster “tree,” but are consistently distinct, as demonstrated in the plot of mds dimension 1 against dimension 4 (Fig. 4C). There are a few Carneddau individuals who appear genetically distinct from the others Carneddau and cluster with the Section A animals or in an intermediate position between the two populations. Structure analysis of the SNPs favoured 6 clusters matching the five sampled populations, but with an additional split found mainly within the Section D cobs (Fig. 5A), a subdivision that is also apparent, though less clearly so, in the mds plots (Fig. 4C). All populations showed significant divergence from each other following pairwise *F*_ST_ analysis of SSR and SNP data (Table 2), with the closest relationship found between the Section As and Carneddau. This is also reflected in Fig. 3, where the Section A and Carneddau form a sub grouping within the tree. Interestingly, for the SSRs, the Section As showed more divergence from the Section Ds (0.0711) compared to the relationship of this group with the other pony breeds, including the Irish Connemara (0.0535) and Scottish Highland (0.0658). This finding is also supported by the data from the SNP analysis, where it is most clearly illustrated by relative positions of the Section A and Section D animals on dimension 1 in the mds plot of dimension 1 versus dimension 4 (Fig. 4C).

**Table 2:** θ_H_ (on the diagonal for SSRs), Pairwise F_ST_ comparisons and effective migration rates between groups based on SSR and SNP data

	Section A	Section D	Connemara	Carneddau	Highland
SSRs					
Section A	**2.013**	3.27	4.42	5.44	3.55
Section D	0.0711	**1.891**	2.76	2.81	1.89
Connemara	0.0535	0.0831	**2.150**	2.68	3.26
Carneddau	0.0439	0.0817	0.0852	**2.055**	2.47
Highland	0.0658	0.1166	0.0711	0.0920	**1.996**
SNPs					
Section A	*	2.44	2.87	4.59	2.35
Section D	0.0931	*	2.69	2.47	2.18
Connemara	0.0802	0.0851	*	3.01	2.60
Carneddau	0.0517	0.0919	0.0766	*	2.47
Highland	0.0960	0.1030	0.0877	0.0918	*

Below diagonal: Pairwise *F*_ST_ based on number of different alleles. Above diagonal: effective number of migrants (Nm) per generation, based on Slatkin 1995. Theta *H* estimates 4N_e_μ = (1/(1−*H*_e_)^2^)−1. All *F*_ST_ values significant at *P* < 0.01.

**Figure 6:**
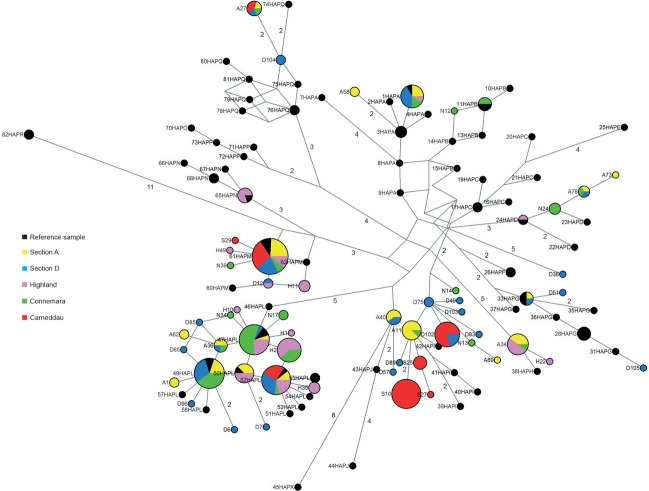
A median-joining network for mtDNA haplotypes based on shared allele frequencies. Node size represents overall haplotype frequency with pie charts within nodes showing frequency of that haplotype by population. Carneddau samples are displayed in red, Section A in yellow, Section D in blue, Connemara in green and Highland in pink. Reference samples representing examples of each of the major haplogroups identified by Achilli et al. (2012) are displayed in black and labelled according to sample number and haplogroup e.g. “1HapA” etc. Numerical values indicate the number of nucleotide changes (>1 mutation) between primary nodes.

Table 3 demonstrates that the maternal lineages in the Section A and Section D animals cannot be distinguished, but that the Carneddau are highly divergent both from these groups and from the Connemaras and Highlands. The Carneddaus had fewer mitochondrial haplotypes present (eight haplotypes within four major haplogroups) compared to other breeds, but contained a number of unique mitochondrial haplotypes present at high frequency within the population (Fig. 6). Thus, Carneddaus may have become isolated from the other groups quite some time ago.

**Table 3:** MtDNA population average pairwise differences

	Section A	Section D	Carneddau	Connemara	Highland
Section A	9.145	9.436	9.977***	8.712***	8.714**
Section D	−0.072	9.871	10.588***	8.874**	9.027
Carneddau	1.540***	1.788***	7.729	10.810***	10.986***
Connemara	0.672***	0.471**	3.478***	6.934	7.377*
Highland	0.499**	0.450**	3.480***	0.268*	7.284

Above diagonal: Average number of pairwise differences between populations (πXY).

Diagonal elements: Average number of pairwise differences within population (πX).

Below diagonal: Corrected average pairwise difference (πXY−(πX + πY)/2).

Probability of result: *<0.05, **<0.01, ***<0.001.

Analyses of potential population expansion based upon Harpending's raggedness score (Harpending 1994) indicated that the Carneddaus were the only population with a highly significant deviation from a unimodal pattern of molecular diversity expected from a population expansion (raggedness index = 0.121, *P* < 0.001), although the Section Ds also had a moderate disturbance (raggedness index = 0.022, *P* < 0.05).

## Discussion

### Population genetic diversity parameters

All groups displayed high levels of mitochondrial haplotype diversity (>0.788 ± 0.0361). SSR/SNP analysis suggested that heterozygosity levels, although lower than some existing reports (Leroy et al. 2009; Prystupa et al. 2012a), remains relatively high and are comparable to values in other European pony breeds, including semi-feral ponies (Solis et al. 2005; Luis et al. 2007; Rendo et al. 2012). Prystupa et al. (2012b) report lower numbers of mitochondrial haplotypes (approximately half) and lower haplotype and nucleotide diversity in Canadian populations of the Connemara, Highland and Welsh Pony than identified here. This perhaps reflects a sampling effect in the founding of those breeds in Canada.

Reduced MTCR haplotype numbers and significant population frequency divergence of the Carneddau could be explained by the effects of severe bottlenecking in a small, recently established but geographically isolated population. However, contrary to expectations for this scenario, mtDNA haplotype diversity remained relatively high and analyses performed to detect evidence of recent potential population expansion demonstrate the Carneddaus have remained at a relatively constant size without any evidence of recent bottleneck contraction in allele diversity. Similarly nuclear diversity is not significantly reduced in the Carneddau, despite the census number of the Carneddau being much lower than the other groups. The Carneddau ponies exist as small, scattered harems across their range and have had minimal human intervention. It appears that this breeding pattern has maintained variation while the population has become genetically isolated via drift or natural selection over an extended period, rather than as a consequence of a recent founder effect. Other free-living ungulate populations have been shown to avoid inbreeding, even in relatively small populations, with a social structure of female philopatry and male-biased dispersal patterns similar to the Carneddau (Coltman et al. 2003; Lawson Handley et al. 2007). In more intensively-managed breeds, popular stallions have artificially large harem sizes, resulting in high variance in male reproductive success, as is apparent in equine pedigree records. Population genetic theory predicts high reproductive variance will dramatically reduce the effective breeding size (Ne) of a population and the similarity in diversity in the Carneddau and other breeds, despite the apparent differences in census numbers, may reflect this effect.

### Population structure and gene flow

The majority of genetic variation between domestic horses and ponies can be attributed to differences between individuals within breeds, where only approximately 8% of variation results from breed differences (Canon et al. 2000; and also found here in this study). However, despite evidence of admixture between these breeds, analysis of population structure using SSR data demonstrated that each population clustered individually according to distinct genotype at the optimal value of *K* = 5. A few Carneddau individuals showed approximately 25%, 50% and 75% assignment to the Section As suggesting recent admixture. This may have occurred if these animals were the first and second generation progeny of three Section A stallions released onto the mountain in 2007–2008, rather than originating from retained similarity to the Section A animals from a common ancestral population. Graziers released Section A stallions at this time to attempt to maintain diversity, because of concerns about the small number of animals. Individuals S17 and S10 in particular cluster with the Section A horses, but do not show a raised level of identity by state with each other, as would be expected if they were siblings by a single stallion. Indeed, despite a lack of knowledge of relationships prior to sampling, the Carneddau do not demonstrate a higher degree of allele sharing than the individuals from the other breeds where siblings had been excluded (Fig. 3). The central position of both the Section A and Carneddau animals in the plot of dimension 2 versus dimension 3 (Fig. 4B) would be consistent with the assumptions that these breeds have retained many ancestral allele frequencies. In contrast the other groups have diverged in different directions as a result of differential selection and specialisation during breed formation. An assessment of the SSR data revealed the presence of an apparently private allele at locus HTG10 in the Carneddau (unique alleles were also observed in the other groups). The 97 bp allele was present in 26% of the Carneddau samples, suggesting that lack of this allele in the other groups is unlikely to be due to under-sampling. Several mitochondrial haplotypes were found to be unique to the Carneddau population, including one predominant within this population. The diversity pattern of the Carneddau showing restricted matrilines combined with more comparable levels of nuclear diversity is unusual. It is possible that the recent admixture of the Section A stallions released into a small population of isolated Carneddau mares could also create the observed diversity patterns. However, the significant differences in allele frequencies required to generate the SNP plots, and unique mitochondrial and SSR alleles present, suggests this is not the case, or at least not the major cause. Therefore, the Carneddau have most likely been isolated for a relatively long period, with effective random breeding and low levels of variability in reproductive success maintaining allelic diversity while allowing change in allele frequencies. As such, the Carneddau appear to be a distinct population that shares an origin with the Welsh ponies in general and more particularly with Section A animals. However, they have been segregated for considerable time to have acquired both allele frequency and mutational changes that are unique and perhaps adapted to their mountain habitat. The recent practice of introducing outside stallions potentially dilutes this distinctive genetic signature.

**Figure 7:**
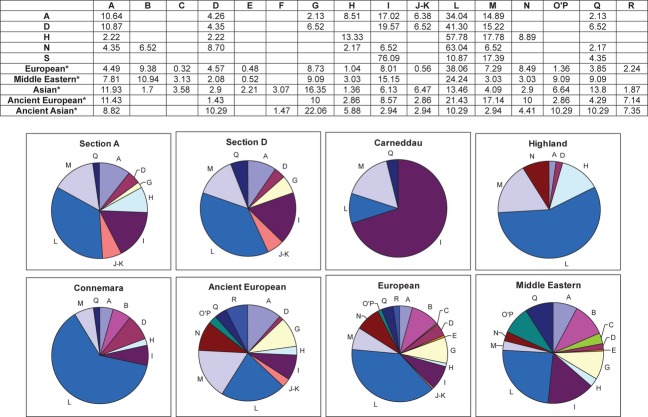
Haplogroup frequency (%) according to breed. The first row identifiers “A” to “R” represent the major worldwide mtDNA haplogroups in horses: haplogroup classification is based upon control region motifs, as described by Achilli et al. (2012). Geographic regions* are according to sequence data collated by Achilli et al. (2012) and comprises of 2401 reference sequences, including ancient (fossil) DNA data.

Mitochondrial sequencing shows extensive sharing of haplotypes across all the populations analysed here and with those from other studies (Achilli et al. 2012; Prystupa et al. 2012b). This is a likely legacy of extensive crossbreeding occurring historically between populations of horses and ponies prior to recognised “closed” breed formation (Vila et al. 2001; McGahern et al. 2006a). However, breed-specific haplogroups were also found (such as haplogroup B in Connemaras and haplogroup N in Highlands. The predominance of haplogroup I in the Carneddau (76% of the population) associated with high autosomal diversity is particularly noteworthy. This haplogroup has not been found above a frequency of 20% in other populations (Achilli et al. 2012) (Fig. 7). Similar over-representation of a particular mitochondrial haplogroup and reduction in haplotype diversity have been demonstrated for the native Irish Kerry Bog Pony (30–50% of population contain Achilli et al. (2012) haplogroup D/Jansen et al. (2002) haplogroup E) where this pattern was taken to indicate the archaic origin of the breed (McGahern et al. 2006b; Prystupa et al. 2012b). The Kerry Bog also shows relatively high nuclear diversity (*H*_O_ = 0.770, Prystupa et al. 2012a). However, the American population of this breed demonstrated low *F*_IS_ values, combined with the lack of any breed-specific clustering following Bayesian analysis. This suggests an effect of significant recent outbreeding and admixture with other pony breeds (supported by historical records, McGahern et al. 2006b). This situation contrasts with the largely population-specific SSR clustering pattern of the Carneddaus, demonstrating that the recent introduction of Section A stallions has not yet resulted in a predominant signature of admixture.

For the mitochondrial data, the Carneddau are the only group consistently significantly different from the other populations, either due to long isolation, or perhaps because they have not been exposed to the non-native mares introduced to “improve” the other breeds. The striking lack of distinction between the Section A and Section D populations in matrilinear diversity suggests that they originate from the same ancestral population of mares. The alternative explanation of extensive interchange of mares is excluded by the clear contrast in the nuclear data for these breeds; the distinct differentiation in the SSR and SNP plots in combination with similarity in mtDNA implies extensive male-biased line-breeding. The Highland and Connemara matrilines were consistently more closely related to each other than to the Welsh ponies. This is again in contrast to the SSR results and dimension 3 of the SNP analysis, which suggest significant gene flow between the Section As and Connemaras: nearly as great as between the Section As and Carneddau. Taken together these results suggest that the maternal and paternal histories of the British upland breeds studied here are quite distinct.

The division within the Section Ds may be consequent on two historic breeding events and subsequent stud management decisions. In the 12th Century Spanish (Iberian) stallions were imported and used to breed with small mountain mares (Davies 1993, 1999). These crosses produced the larger “Powys Horse” which may form one of the founding types of the Section D ponies. In the 19th century livestock drovers brought fashionable trotters/Hackneys from the East of England to breed with local Welsh mares (Welsh Pony and Cob Society 2002). Regional studs within Wales may have acquired different levels of the incoming genes from these two periods and divergence in breeding target between studs maintained the genetic dichotomy within the Welsh Section Ds, even after the closure of the stud book.

### Implications for conservation of Carneddau ponies

Although clearly related to the Section A pony, the Carneddau represent a genetically distinct population. For many populations of domestic livestock the definition of uniqueness is relatively subjective and differences between two populations may only be a function of the relative frequencies of a few genes, often associated with a single physical character or small group of specific characters (Henson 1992; Groenveld et al. 2010). Any population that is historically, geographically or reproductively isolated (has had little genetic influence from other breeds for a long time period) could be considered to be a unique population (Henson 1992). The Carneddau ponies may therefore represent an important source of uncharacterized genetic diversity and adaptations restricted to less intensively managed upland populations. In this study we have detected more than 10 times the number of SNP loci expected with extreme deviations from HW equilibrium after Bonferroni correction for the number of tested loci. These thereby form a potential set of markers for determining the genetic differentiation between the groups as a consequence of drift or selection, and as a future target for understanding adaptations to the upland environment. Specific adaptations have been found in upland and moorland populations of feral or free-living species previously, including parasite load tolerance (Coltman et al. 1999) and coat hair physiology (Wilson 2011). The importance of preserving feral/wild populations as valuable sources of genetic diversity for domestic relatives has been highlighted in other species (Schoen and Brown 1993; Giovambattista et al. 2001; Tapio et al. 2005; Taberlet et al. 2008; Medugorac et al. 2009). The value of hardy native pony breeds for encouraging biodiversity and repopulation of rare species has also been recognized within conservation grazing schemes (Mitchell and Kirby 1990; Kohyani et al. 2008). The semi-feral Carneddau ponies play a vital role within the mountain ecology of the Snowdonia National Park, as they are involved in a grazing scheme maintaining preferred habitat for an endangered bird, the red-billed chough (Pyrrhocorax pyrrhocorax) (Murray 2006; Johnstone et al. 2010). Limited funding was provided for the land owners to manage a subgroup of ponies upon the open mountain; however the project has now ended.

The Carneddau population is believed to be ≤300 individuals and at risk due to economic pressures on the land they graze. A derogation has been granted so that only ponies sold at auction have to be passported and microchipped, rather than requiring that all new-born Carneddaus are issued with a passport (in accordance with UK law for other equids). However, because the ponies are unregistered their sales value is very low (typically a meat value), and so the expense of microchipping and passporting is unviable for their owners. Animals which are sold have limited use; they have no pedigree and are not registered as a recognised breed, preventing them from competing in many show classes. From the perspective of population conservation, the removal of young fillies for auction may compound the issue of reduced mitochondrial haplotype numbers. Other human selection practices are minimal in these animals, making them a particularly important group. In the New Forest, Dartmoor and the Brecon Beacons, specially selected individual stallions are released every year into known, limited geographical areas frequented by free-living mares and re-captured at the end of the breeding season. With the exception of three stallions released (but only one since removed) in 2007–2008, this does not occur with the Carneddau population.

It is widely acknowledged that domestic pony and horse breeds have undergone varying degrees of admixture and gene flow prior to establishment of defined, “closed” pedigrees (Thirstrup et al. 2008; Groenveld et al. 2010; Prystupa et al. 2012a). This is clearly evident in the genetic data presented here, for all of the populations considered. However, in terms of *relative* levels of admixture, the Carneddaus appear to show similar degrees to that found between the recognised breeds included in this analysis. In addition to this, the distinct genetic signatures (mtDNA haplotypes, SSR frequencies, potential SNP adaptations) suggest that the Carneddau ponies are a defined, long-established population worthy of conservation. However, whilst the WPCS register Section A ponies that are free-living on the mountains of south Wales, this semi-feral grouping does not include the Carneddau. Consequently, the Carneddau are excluded from current Rare Breeds Survival Trust conservation management schemes and are not considered for use within nationwide conservation grazing projects utilising ponies such as Dartmoor, Exmoor and imported Polish Koniks. The current lack of recognition surrounding the Carneddau ponies contributes to the risk of losing a potentially useful, and distinct, native British breed.

## Conclusions

The genetic relationship of Carneddau ponies in relation to other native breeds of the UK and Ireland was revealed by the integrated results of each genetic analysis. The Carneddau ponies have a shared ancestry with the recognized Section A pony yet have remained in relative isolation, subjected to natural selective pressures for a significant period of time. This has been followed by more recent genetic introgression by Section A stallions and round up practices. The genetic impact of human activities, particularly the selective use of stallion crossing upon the pony breeds, are evident in the comparison of the different genetic systems; both more recently in the effect upon SSR frequencies within the Carneddaus and historically in the SNP profile of the Section D cobs. Despite the recent management practices, the Carneddau ponies maintain a distinct genetic signature that should be conserved. Given the restricted geographic distribution and the low numbers of the Carneddau, in order to provide some conservation protection measures, these animals should be classified as a rare pony population at critical extinction status. This study illustrates the relevance and potential power of combining autosomal and maternally transmitted polymorphic assays to unravel genetic structure and relatedness in feral and intensively managed populations. Herein we provide new information and knowledge to support conservation strategies of endangered breeds.

## Appendix

Figure A1. The results of STRUCTURE analysis for SSR loci of each population (including admixed Section B and Section C) for *K* = 5. Populations are labelled as follows: A = Section A, B = Section B, C = Section C, D = Section D, S = Carneddau, N = Connemara and H = Highland.
